# Evaluation of care for people with HIV in Primary Health Care: construct validation

**DOI:** 10.1590/0034-7167-2023-0190

**Published:** 2025-01-13

**Authors:** Clarissa Mourão Pinho, Juliana da Rocha Cabral, Morgana Cristina Leôncio de Lima, Mônica Alice Santos da Silva, Regina Celia de Oliveira, Jael Maria de Aquino, Erika Simone Galvão Pinto, Maria Sandra Andrade

**Affiliations:** I Universidade de Pernambuco. Recife, Pernambuco, Brazil; II Universidade Federal do Rio Grande do Norte. Natal, Rio Grande do Norte, Brazil

**Keywords:** HIV, Acquired Immunodeficiency Syndrome, Program Evaluation, Primary Health Care, Comprehensive Health Care, VIH, Síndrome de Inmunodeficiencia Adquirido, Evaluación de Programas y Proyectos de Salud, Atención Primaria de Salud, Atención Integral de Salud

## Abstract

**Objectives:**

to verify the construct validation of an instrument for evaluating care for people living with HIV in Primary Health Care.

**Methods:**

methodological study carried out in 2021 with 260 health professionals in Recife, PE. Validation based on the internal structure was carried out at this stage using exploratory and confirmatory factor analysis, and validity based on item response theory.

**Results:**

the validation determined the retention of five factors and 63 items. The instrument’s internal consistency and quality of fit was 0.90, the Tukey-Lewis index was 0.915 and the comparative fit index was 0.918 in the confirmatory factor analysis. The indication for the absolute majority of items is adequate fit.

**Conclusions:**

the instrument has construct validity, making it possible to use it to evaluate the decentralization process and care for People Living with HIV in Primary Health Care.

## RESUMEN

**Objetivos:**

comprobar la validez de constructo de un instrumento de evaluación del cuidado a personas que conviven con VIH en la Atención Primaria de Salud.

**Métodos:**

estudio metodológico realizado en 2021 con 260 profesionales de salud en Recife, Pernambuco. La validación se realizó con base en la estructura interna; se utilizó el análisis factorial exploratorio y confirmatorio, así como la validez según la teoría de respuesta al ítem.

**Resultados:**

la validación determinó la retención de cinco factores y 63 ítems. La consistencia interna y la calidad de ajuste del instrumento fueron de 0,90, el índice de Tukey-Lewis, de 0,915 y el índice de ajuste comparativo, de 0,918 en el análisis factorial confirmatorio. La mayoría de los ítems mostró ajuste adecuado.

**Conclusiones:**

el instrumento presenta validez de constructo y es propicio para evaluar el proceso de descentralización y de cuidado a personas con VIH en la Atención Primaria de Salud.

## INTRODUCTION

The proposal for the new model of comprehensive care for people living with HIV (PLHIV) considers that Primary Health Care (PHC) can play a decisive role in shared care with specialized services. Until then, the health care model was centered on Specialized Care Services (SCS). The adoption of the new care system makes it possible to expand diagnosis through rapid testing and timely access to antiretroviral drugs (ARVs). In addition, PHC is expected to stratify individuals into symptomatic and asymptomatic, carry out routine tests such as TCD4 lymphocyte count and viral load (VL), and provide professional training. Co-infected people – pregnant women, children and individuals with some resistance to ARVs – will be referred to the SCS, so that the latter will provide matrix support for the care provided at the PHC^(1)^.

The adoption of the model is based on the assumption that PHC care is fundamental to improving management, infection control and health care for PLHIV. This practice can provide care focused on individual needs, promote the bond between professional and patient and optimize actions aimed at promotion, prevention, diagnosis, treatment and health education^(1,2,3)^. In order to operationalize or maintain this system of care, it is necessary to monitor and evaluate the functioning of the health care system, with the definition and structuring of the Health Care Network (RAS) and its respective flows for the care of these users^(2)^.

The use of a validated evaluation tool makes it possible to enhance capacities and improve care, since systematic evaluations produce cycles of improvements that promote organizational learning. From this perspective, evaluation should be used as a means of identifying opportunities for improvement, helping to propose solutions with a view to using resources more efficiently and effectively, as well as reorganizing health practices within a political, economic, social and professional context^(4,5)^. Carrying out evaluation cycles enables managers and professionals to develop systemic thinking about the performance of the care network for people living with HIV and contributes to continuous improvement in the quality of health care^(6)^.

With the aim of collaborating with the process of decentralizing care for PLHIV to PHC, after validating the content of the instrument “Evaluation of the process of decentralizing care for People Living with HIV to Primary Health Care”^(7)^ construct validation was carried out. Validated evaluation tools enable a systemic view of structure, process and results and guide decisions to improve the quality of healthcare, especially the actions carried out in PHC^(8,9)^. Consistent and well-founded analyses allow for the improvement of capacities, greater resolutiveness, quality of assistance and comprehensive care^(4,5)^.

Construct validation is considered the main evaluation measure of an instrument, as it is through this stage that it is possible to verify the degree to which the instrument measures what it set out to measure. It is hoped that the instrument, in its final version, will allow health professionals and managers to see beyond the complexity of the details of the decentralization process, and identify the implicit structures that can contribute to effective care for people living with HIV. It is also hoped to contribute to improving health services in which the decentralization process is already in place, as well as helping to structure health units that have not yet joined the decentralization process.

## OBJECTIVES

To verify the construct validation of an instrument for evaluating care for people living with HIV in Primary Health Care.

## METHODS

### Ethical aspects

The ethical precepts of Resolution 466/2012 were respected. The research project was approved by the Research Ethics Committee of the Oswaldo Cruz University Hospital (HUOC)/*Pronto Socorro Cardiológico Universitário de Pernambuco –* Prof. Luiz Tavares (PROCAPE).

### Study design, period and location

Methodological study to validate the construct of a health evaluation tool. The study is part of the project“Construction and validation of an instrument to evaluate the process of decentralizing care for HIV patients to Primary Health Care”. The standards presented by the American Educational Research Association (AERA), the American Psychological Association (APA) and the National Council on Measurement in Education (NCME) were taken into account, as well as the best practices recommended for construct validation^(10,11)^.

Data was collected from August to November 2021 in 103 health centers, distributed between the Family Health Strategy (FHS), Primary Care Center (PCC) and Community Health Workers Program (CHWP) in the municipality of Recife, Pernambuco, Brazil.

### Population or sample; inclusion and exclusion criteria

The study population consisted of health professionals working in PHC. To define the sample, during data collection, the Kaiser-Meyer-Olkin (KMO) measures of sampling adequacy of the instrument were calculated, followed by the Measure of Sampling Adequacy (MSA) per item. The reference value for KMO was a minimum of 0.70 and for MSA a minimum of 0.50 for each item^(11)^, the number of interviewees (n=260) presented satisfactory results for the validation of the construct^(11)^. The inclusion criteria were: being a senior health professional (doctor, nurse or dentist) and having worked in the FHS, PCC or CHWP for at least one year. We excluded mid-level health professionals and those in higher education who were not directly involved in care at the units studied.

### Study protocol

The instrument was evaluated using a Likert-type scale, with the following options: Never, Sometimes, Often, Very Often and Always.

For data collection, the day and time were scheduled in advance, according to the availability of the health professionals, which took place in person at the health center. The data was collected individually, in a room at the center. After signing the Free and Informed Consent Form and providing guidance on the research, the interviews were carried out.

### Analysis of results and statistics

Data analysis was carried out in two stages: 1) validity based on internal structure, which is related to statistical analysis considering models for latent trait measures. In this stage, exploratory factor analysis (EFA) and confirmatory factor analysis (CFA) were used; and 2) validity based on the pattern of response to the items, which refers to theoretical and empirical evaluations of how the participants responded to the instrument. For this study, we used analyses based on item response theory such as differential item functioning^(11)^.

To carry out the EFA, the measures of sampling adequacy of the instrument were first calculated using the KMO and then the MSA per item was calculated^(11)^. For Bartlett’s test of sphericity, p-values ≤ 0.05^(12)^ were adopted. The method used to carry out the EFA was polychoric factor analysis, called Weighted Least Squares (WLS). Internal consistency was verified using Cronbach’s alpha, with values equal to or above 0.70 for the instrument to be considered accurate/reliable^(13)^.

The protocols followed to interpret the results of the EFA were the following indices and values: the communality (h2) referring to the measure of the inter-item relationship; the eigenvalue, used as a criterion to define the number of factors^(14)^; the scarp graph, known as a screeplot, interpreted using the Cattell criterion, also used to define the number of factors^(15)^; and the factor loadings of the items, measured in order to verify which items are allocated to which factors, the factor loadings were set at ±0.30^(11)^.

To carry out the CFA, the principles of structural equation modeling (SEM) were used. The following indicators were analyzed: χ^2^ (chi-square); Comparative Fit Index (CFI) and Tucker-Lewis Index (TLI); Godness-of-Fit Index (GFI) and Adjusted Goodness-of-Fit Index (AGFI); Root-Mean-Square Error of Approximation (RMSEA)^(16,17)^.

For χ^2^, 3 was taken as the maximum value to indicate that the theoretical model is adjusted to the data. For CFI and TLI, scores higher than 0.90 were used to affirm that the theoretical model best represents the construct. For the GFI and AGFI, values greater than 0.90 were used and for the RMSEA values ≥ 0.05, although it should be noted that for larger samples a value of 0.08 is acceptable^(16,17)^.

In order to analyze the validity based on the response pattern to the items, the item response theory (IRT) was applied. In the case of this study, the data is polytomous or categorical, so it is necessary to apply a model for graded responses, so in the case of this study, Samejima’s graded response model was applied, which investigates discrimination, ranging from 0 to +3, the higher the more discrimination, and difficulty on ordinal or categorical scales, ranging from -3 to +3, the easier and more difficult, respectively^(18,19)^.

## RESULTS

The results will be presented according to the following steps: exploratory factor analysis, confirmatory factor analysis and item fit analysis – item response theory.

### Exploratory Factor Analysis

The sample adequacy assessment showed satisfactory results (KMO=0.786). Bartlett’s Test of Sphericity was also significant [χ^2^(62) = 2446.8, p-value <0.001]. The adequacy of the items verified by the KMO showed results higher than 0.50, except for the items: “Have you ever run out of male condoms at the unit?”(MSA = 0.498); “Have you ever run out of female condoms at the unit?” (MSA = 0.491);“Does the Family Health Unit provide care for pre-exposure prophylaxis (PrEP)?” (MSA = 0.483); and “Does the Family Health Unit provide care for post-exposure prophylaxis (PEP)?” (MSA = 0.483). However, as these values were considered marginal, it was decided to maintain them in the following analyses.

**Figure 1:**
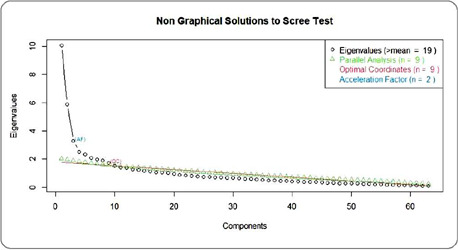
Comparative chart for choosing the number of factors for the instrument, Recife, Pernambuco, Brazil, 2022

Factor analysis using the WLS method showed that the Kaiser criterion, based on the number of Eigenvalues (eigenvalues), suggested 19 factors, while the Cattell criterion, based on the elbow point, identified in the graph as Optimal Coordinates, indicated 9 factors. The Parallel Analysis criterion, Horn’s criterion, also indicated 9 factors, and the Acceleration Factor criterion indicated 2 factors for the instrument (Figure 1).

In the process of deciding on the factors, considering their factor loadings, EFAs were carried out for structures containing 9 factors, as indicated by the parallel analysis and Optimal Coordinates methods, as well as for the bifactor structure as indicated by the acceleration factor. However, both structures were unsatisfactory in theoretical terms. Therefore, two more versions were tested with 7 factors suggested by the expert judges and with 5 factors suggested by the technical reviewers in the content validation^(7)^.

With regard to the analysis carried out between factors and items, it is stated that the item belongs to a given factor if it has values between -0.3 and ≥0.3^(20,21)^. When the factors and their respective items were distributed, it was observed that in the 9-factor option, only 4 items did not factor. In the 7-factor option, 3 items did not factor. In the 5-factor option, 8 items did not factor, and in the 2-factor option, 16 items did not factor.

It was decided that the structure that showed the best suitability in statistical and theoretical terms was the structure with 5 factors. Thus, the factors were renamed WLS1 – Surveillance, prevention and Health Care Networks; WLS2 – Support, diagnosis and reception; WLS3 – Health education; WLS4 – Organization of care; and WLS5 – Physical, material and human resources and the items relocated according to each factor.

Although items 1, 4, 5, 6, 7, 19, 52 and 54 did not show initial results within the statistical recommendations, it was decided to keep the items in the instrument for theoretical reasons. The items were then relocated to the factor they best fit, except for items 19 and 54, which were removed from the analysis after consideration of their factor loadings and their support for the statistical model. It should be noted that although items 19 and 54 do not factor, they were not excluded due to their theoretical importance, and it is suggested that these items be applied in future studies. Thus, item 19 was allocated to factor WLS5 Physical, material and human resources and item 54 to factor WLS4 – Organization of care.

### Confirmatory Factor Analysis

According to the results, the instrument has indicative values for composite reliability (0.99) and average variance extracted (0.99) in all the associations made between the factors and their respective items. Furthermore, it is clear that for all the associations the p-value was significant (p-value <0.05), which is sufficient to consider this instrument valid and accurate. The Structural Equation Modeling (SEM) fit indicators for validating the instrument are shown in Table 1. The criteria follow the interpretation explained in the methodology, regarding the indicators needed to fit the model to the data. The structure of the instrument is considered valid according to the indicative criteria. It should be noted that items 19 and 54 were not considered for the CFA, given the decisions made regarding the statistical and theoretical support for keeping these items.

**Table 1:** Adjustment indicators of the Confirmatory Factor Analysis for construct validation of the instrument for evaluating the process of decentralization of care for People Living with HIV for Primary Health Care, Recife, Pernambuco, Brazil, 2022

Adjustment indicator	Criteria for a good model fit	Final model
Absolute adjustment		
Discrepancy function: χ^2^ (p-value)	-	2749.959 (0.001)
Normed chi-square (χ^2^/gl)	Value between 1 and 5	1.56
GFI (goodness of fit index)	Above 0.90	0.90
AGFI (adjusted goodness of fit index)	Above 0.90	0.90
RMSEA (root mean square error of approximation)	Between (0.05;0.10] p(H0: RMSEA≤0.05)	0.047
Relative adjustment		
TLI (Tukey-Lewis index)	≥0.90	0.915
CFI (Comparative fit index)	≥0.90	0.918

### Item Fit Analysis – Item Response Theory

The analysis model was decided by means of the ANOVA test, comparing the Generalized Partial Scale Model and the Generalized Partial Scale Model^(22)^ and Samejima’s Graded Response Model^(19)^. According to the results, the model chosen was Samejima’s graded response model with a significant ANOVA result [χ2(0) = 216.078; p-value < 0.01].

It should be noted that, in this case, no significant p-values are expected. Although items 19 and 54 were not considered in the CFA, they showed good fit indices and could be kept in the instrument for future research. On the other hand, the items “Does the Family Health Unit offer rapid HIV testing for all pregnant women being monitored at the unit?”(p-value =0.030); “Does the Family Health Unit offer rapid HIV testing for individuals diagnosed with tuberculosis?”(p-value=0.000);“Does the Family Health Unit offer antiretroviral drugs for HIV treatment?“ (p-value=0.043); and the item “Do the Medicines Dispensing Units (MDU) care for HIV patients referred by the Family Health Unit?”(p-value=0.28), showed significant p-value results, indicating that these items were not adjusted for validation based on the parameters of the items. It can also be seen that the RMSEA value was less than 0.05 for all the items, except item 40 (0.07), which was still less than 0.08.

**Figure 2:**
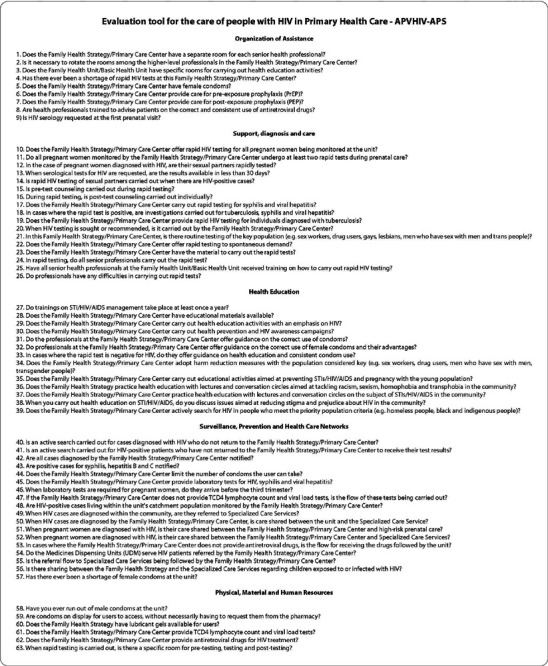
Final version of the instrument for evaluating the process of decentralizing care for People Living with HIV in Primary Health Care, with five factors and sixty-three items, Recife, Pernambuco, Brazil, 2022

Therefore, the indication for the absolute majority of the items is that the fit is adequate. Thus, in its final version, the 63 items were organized according to their factor. Each item should be evaluated using a Likert-type scale, with the following options: Never, Sometimes, Often, Very Often and Always (Figure 2).

## DISCUSSION

Construct validation, considered to be the main evaluation measure of an instrument, was carried out using EFA, CFA and item fit analysis, with IRT^(23,24)^. The importance of using scientific knowledge to build reliable tools for decision-making in health services is highlighted. The adoption of these tools boosts quality gains and makes it possible to improve care for people living with HIV in Primary Health Care.

This makes it easier to measure the actions taken by the manager to improve the process. Thus, the use of the tool by multidisciplinary teams and managers allows them to cooperate with each other to achieve a certain goal. It also enables systemic thinking about the care network for people living with HIV and helps professionals, managers and individuals to realize that they are all part of a single system that seeks continuous improvement in the comprehensive care model for PLHIV^(6,7,8)^.

The results of the analysis of the instrument were satisfactory in terms of the adequacy of the sample and the correlation/ covariance matrix for almost all the items, except for four items, which were not excluded, since all the participating health professionals declared “never”as their answer to the items, i.e. there had never been a shortage of male and female condoms in the units investigated. In addition, the items are within the recommendations of the Ministry of Health and, for this reason, it was decided not to exclude the items. The items dealing with the provision of Pre-Exposure Prophylaxis (PrEP) and Post-Exposure Prophylaxis (PEP) were also kept because they are actions that should be carried out in PHC^(25)^.

In the proposal for the new model of comprehensive care for people living with HIV, the concept of combined prevention stands out, which is configured as a set of behavioral, biomedical and structural measures and involves different levels of care. Thus, the use of male and female condoms is the main barrier method for the prevention of HIV and STIs. It is a cheap, easily accessible and effective method, and it is recommended that it be offered widely and without access barriers^(26,27)^.

PrEP is currently considered one of the most innovative HIV prevention strategies and should be widely disseminated in PHC. It can be seen that acceptance of PrEP is still low, due to some barriers encountered at this level of care, such as: lack of funding, difficulty of access, stigma, lack of training and knowledge on the part of health professionals, among others^(28,29)^. Decentralizing care and prescribing PrEP could be an alternative for expansion. In a recent study carried out in Canada, there was an increase in adherence to the measure when carried out by nurses^(30)^. PEP is another recommended strategy, its use has been increasing in Brazil and worldwide, and is recommended in situations of accidents with biological materials, sexual violence, as well as consensual sexual exposure^(31)^.

It can be seen that the 5-factor option was not the alternative with the lowest number of items that did not factor, but this option was chosen because the items had similar aspects. The aim of EFA is to determine the number of latent variables (factors) that best represent a number of observed variables (items), i.e. the observed variables are assigned to the same factor when they have a common variance^(32)^. EFA requires researchers to make a series of decisions in order to obtain an adequate factor structure (the relationship between the factor and the items), and these decisions need to be anchored in theoretical and methodological criteria^(14)^.

Regarding the items that did not factor in, items 1 and 4 are related to the provision of rooms for individualized care by senior health professionals; items 5, 6, 7, 8 and 19 explore aspects related to the presence, absence and access to supplies, such as rapid tests and condoms. Items 52 and 54 investigate the training of professionals in rapid testing and guidance on the use of ARVs.

Therefore, it is recommended that adequate physical spaces be made available to ensure patient confidentiality and privacy during reception, counseling, testing and health education activities, among others^(27)^. It should be emphasized that health education practices go beyond the transmission of knowledge. These practices should promote the empowerment of individuals and the population, since through information it is possible to change attitudes and adopt practices that are more consistent with STI/HIV/AIDS prevention strategies^(33,34)^. It should be noted that the health professionals who will make up the RAS for PLHIV should be provided with matrix support by the SCSs, and it is also recommended that these professionals be trained through supervised internships, shared consultations and conversation circles, for example^(1,29)^.

With regard to the CFA, the instrument showed satisfactory levels of composite reliability and average variance. With regard to the CFA fit indicators, the instrument met the criteria for a good model fit, with satisfactory RMSEA, GFI and CFI. The fit index is a measure that helps verify how good the instrument is and in this case, the instrument met all the criteria and was considered validated^(9)^.

It is worth noting that decentralizing care for PLHIV to PHC is a possibility that would expand access to health services for PLHIV, with increased testing, diagnosis and timely treatment, as well as prevention of HIV infection through the use of PrEP and PEP. However, there are still several obstacles to the success of this proposal^(35,36)^, such as the lack of a physical structure for counseling and rapid testing, lack of professional training, materials and supplies, such as educational materials such as folders, posters, female condoms, which reflects negatively on the quality of care^(37)^.

Given this context, it can be seen that despite the Ministry of Health’s recommendations to decentralize care for people living with HIV to PHC, there is a need for greater investment in this level of care, structuring units, defining access flows, providing materials and supplies, and guaranteeing an adequate number of trained human resources. Some strategic actions have been adopted in PHC, such as: activities aimed at welcoming and counseling, offering rapid testing and serological tests for HIV, syphilis and viral hepatitis, making condoms and lubricant gels available, active search, notification and health education practices^(35,36,37,38,39)^ which could be the way to fully implement the new model of care for people living with HIV.

Health management quality assessment tools are the cyclical basis of management dynamics for improving processes. They enable learning cycles and the implementation of action plans or the standardization of practices in a systemic way and with the participation of the multi-professional team. In order to apply the tool more effectively, it is necessary to take into account the digital transformation in health and adherence to technologies that enable greater efficiency in care, cost reduction, standardization of the quality of the physical and functional structure, processes and results. The incorporation of this evaluation tool into platforms that can be developed in future research, or which already exist in PHC, will streamline health management evaluation processes and contribute to faster and more effective decision-making.

### Study limitations

A limitation of the study is the fact that it was carried out in just one municipality in the Northeast. In addition, the sample collected was used to validate the construct, and it is recommended that the study be applied to a larger sample, including other services, to enable the scores for the factors to be validated.

### Contributions to the fields of nursing, health or public policy

It is hoped that the validated instrument will contribute to the evaluation of the process of decentralizing care for PLHIV in PHC and to the structuring/restructuring of units, since PHC has the potential to provide this care. In addition to contributing to health professionals, especially nurses who provide care to PLHIV, in verifying essential aspects for the implementation/consolidation of the proposed new model of care for people living with HIV.

## CONCLUSIONS

The instrument for evaluating care for People Living with HIV for Primary Health Care has been validated in terms of its construct according to the recommendations of the AERA, APA and NCME institutions. In its final version, it has 63 items, divided into 5 factors: Organization of care; Support, Diagnosis and Reception; Health Education; Surveillance, Prevention and Health Care Networks; Physical, Material and Human Resources, assessed using a Likert-type scale.

It is hoped that the instrument will help in health evaluation, as well as in the structuring and/or restructuring of health units with a view to providing comprehensive, quality care to PLHIV in PHC. In addition, it is believed that the use of this tool will make it possible to evaluate the implementation of actions aimed at decentralizing care for PLHIV to PHC, as well as identifying the potential and weaknesses of the process, with a view to quality of care and comprehensive care for PLHIV. Further studies are recommended with the aim of standardizing the scores of the validated instrument, as well as incorporating the instrument into digital platforms.
