# Supplementary material for: Evaluation of care for people with HIV in Primary Health Care: construct validation
Source: Rev Bras Enferm. 2025 Jan 13;77(6):e20230190. doi: 10.1590/0034-7167-2023-0190 (PMC11731853; doi:10.1590/0034-7167-2023-0190)
Supplement: Translation

# Translation: Avaliação da assistência a pessoas com HIV na Atenção Primária à Saúde: validação de constructo

**DOI:** 10.1590/0034-7167-2023-0190pt

**Authors:** Clarissa Mourão Pinho, Juliana da Rocha Cabral, Morgana Cristina Leôncio de Lima, Mônica Alice Santos da Silva, Regina Celia de Oliveira, Jael Maria de Aquino, Erika Simone Galvão Pinto, Maria Sandra Andrade

**Affiliations:** I Universidade de Pernambuco. Recife, Pernambuco, Brasil; II Universidade Federal do Rio Grande do Norte. Natal, Rio Grande do Norte, Brasil

**Keywords:** HIV, Síndrome da Imunodeficiência Adquirida, Avaliação de Programas e Projetos de Saúde, Atenção Primária à Saúde, Assistência Integral à Saúde

## Abstract

**Objetivos:**

verificar a validação de constructo de um instrumento de avaliação da assistência a pessoas vivendo com HIV na Atenção Primária à Saúde.

**Métodos:**

estudo metodológico, realizado em 2021 com 260 profissionais de saúde, em Recife, PE. Realizou-se a validação baseada na estrutura interna, nesta etapa, utilizou-se a análise fatorial exploratória e confirmatória, e a validade baseada na teoria de resposta ao item.

**Resultados:**

a validação determinou a retenção de cinco fatores e 63 itens. A consistência interna do instrumento e qualidade do ajustamento foi de 0,90, o índice de Tukey-Lewis foi de 0,915 e o índice de ajuste comparativo foi de 0,918 na análise fatorial confirmatória. A indicação para a maioria absoluta dos itens é de ajuste adequado.

**Conclusões:**

o instrumento apresenta validade de constructo, possibilitando a utilização para avaliação do processo de descentralização e da assistência às Pessoas Vivendo com HIV na Atenção Primária à Saúde.

## INTRODUÇÃO

A proposta do novo modelo de cuidado integral às pessoas vivendo com HIV (PVHIV) considera que a Atenção Primária à Saúde (APS) pode desempenhar papel decisivo no cuidado compartilhado com os serviços especializados. Até então, o modelo de atenção à saúde estava centrado nos Serviços de Assistência Especializada (SAE). A adoção da nova sistemática de assistência possibilita a ampliação do diagnóstico, através da testagem rápida e do acesso oportuno aos antirretrovirais (ARV). Além disso, é previsto que na APS seja realizada a estratificação dos indivíduos em sintomáticos e assintomáticos, exames de rotina, como contagem de linfócitos TCD4 e carga viral (CV) e a qualificação profissional. As pessoas co-infectadas – gestantes, crianças e indivíduos com alguma resistência aos ARV – serão referenciadas para os SAE, de forma que este último dará suporte matricial aos atendimentos realizados na APS^(1)^.

A adoção do modelo parte do pressuposto que a assistência na APS é fundamental para a melhoria da gestão, para o controle da infecção e para a atenção à saúde de PVHIV. Tal prática pode proporcionar uma assistência centrada nas necessidades individuais, promover o vínculo entre profissional e paciente e a otimização das ações voltadas à promoção, prevenção, diagnóstico, tratamento e educação em saúde^(1,2,3)^. Para operacionalização ou manutenção dessa sistemática de assistência, é necessário o acompanhamento e a avaliação do funcionamento do sistema de atenção à saúde, com a definição e a estruturação da Rede de Atenção à Saúde (RAS) e seus respectivos fluxos para a assistência a esses usuários^(2)^.

A utilização de um instrumento de avaliação validado possibilita o aprimoramento de capacidades e melhorias na assistência, uma vez que as avaliações sistemáticas produzem ciclos de melhorias que promovem o aprendizado organizacional. Nessa perspectiva, a avaliação deve ser utilizada como um meio para a identificação de oportunidade de melhorias, contribuindo para propor soluções com vistas à utilização dos recursos de modo mais eficiente e eficaz, bem como à reorganização das práticas em saúde, dentro de um contexto político, econômico, social e profissional^(4,5)^. A realização de ciclos de avaliação possibilita aos gestores e profissionais o desenvolvimento do pensamento sistêmico sobre a atuação da rede de atenção às pessoas vivendo com HIV e contribui para melhoria contínua da qualidade do atendimento à saúde^(6)^.

Com o objetivo de colaborar com o processo de descentralização da assistência às PVHIV para a APS, após a validação de conteúdo do instrumento “avaliação do processo de descentralização do atendimento a Pessoas Vivendo com HIV para Atenção Primária à Saúde”^(7)^, foi realizada a validação de constructo. Instrumentos de avaliação validados possibilitam a visão sistêmica da estrutura, do processo e do resultado e direcionam as decisões para a melhoria da qualidade da assistência em saúde, em especial, para as ações desenvolvidas na APS^(8,9)^. Análises consistentes e fundamentadas permitem o aprimoramento de capacidades, maior resolutividade, qualidade da assistência e integralidade do cuidado^(4,5)^.

A validação de constructo é considerada a principal medida de avaliação de um instrumento, pois é por meio desta etapa que é possível verificar o grau com que o instrumento mede o que se propôs a mensurar. Espera-se que o instrumento, em sua versão final, permita aos profissionais de saúde e gestores enxergarem além da complexidade dos detalhes do processo de descentralização, que possibilite identificar as estruturas implícitas que podem contribuir para a efetividade da assistência às pessoas vivendo com HIV. Espera-se, ainda, contribuir para melhoria dos serviços de saúde nos quais o processo de descentralização já esteja instalado, como também auxiliar na estruturação das unidades de saúde que ainda não aderiram ao processo de descentralização.

## OBJETIVOS

Verificar a validação de constructo de um instrumento de avaliação da assistência a pessoas vivendo com HIV na Atenção Primária à Saúde.

## MÉTODOS

### Aspectos éticos

Foram respeitados os preceitos éticos da Resolução 466/2012. O projeto de pesquisa foi aprovado pelo Comitê de Ética e Pesquisa do Hospital Universitário Oswaldo Cruz (HUOC)/Pronto Socorro Cardiológico Universitário de Pernambuco – Prof. Luiz Tavares (PROCAPE).

### Desenho, período e local do estudo

Estudo metodológico de validação de constructo de instrumento de avaliação em saúde. O estudo integra o projeto “Construção e validação de um instrumento de avaliação do processo de descentralização do atendimento ao paciente com HIV para Atenção Primária à Saúde”. Foram consideradas as normas apresentadas pelas instituições *American Educational Research Association* (AERA), *American Psychological Association* (APA) e *National Council on Measurement in Education* (NCME), e as melhores práticas recomendadas para validação de constructo^(10,11)^.

Os dados foram coletados no período de agosto a novembro de 2021, em 103 unidades de saúde distribuídas entre Estratégia de Saúde da Família (ESF), Unidade Básica de Saúde (UBS) e Programa de Agentes Comunitários de Saúde (PACS), no município de Recife, Pernambuco, Brasil.

### População ou amostra; critérios de inclusão e exclusão

A população do estudo foi composta por profissionais de saúde atuantes na APS. Para definição da amostra, durante a coleta dos dados, calculou-se as medidas de adequação amostral do instrumento através do Kaiser-Meyer-Olkin (KMO) e, posteriormente, foram calculadas a *Measure of Sampling Adequacy* (MSA) por item. Utilizou-se como valor de referência para KMO resultados mínimos de 0,70; para MSA o valor mínimo foi de 0,50 para cada item^(11)^, constatando-se que o número de entrevistados (n=260) apresentava resultados satisfatórios para a validação do constructo^(11)^. Utilizou-se como critério de inclusão: ser profissional de saúde de nível superior (médico, enfermeiro e odontólogo) e atuar na ESF, UBS, PACS, há pelo menos 1 ano. Excluiu-se os profissionais de saúde de nível médio e aqueles de nível superior que não atuavam diretamente na assistência nas unidades estudadas.

### Protocolo do estudo

A avaliação do instrumento foi realizada utilizando uma escala do tipo *Likert*, com as seguintes opções: Nunca, Algumas vezes, Frequentemente, Muito frequentemente, e Sempre.

Para a coleta de dados, realizou-se o agendamento prévio do dia e horário, de acordo com a disponibilidade dos profissionais de saúde, que ocorreu de forma presencial na unidade de saúde. Os dados foram coletados de forma individual, em sala da própria unidade. Após a assinatura do Termo de Consentimento Livre e Esclarecido e orientações sobre a pesquisa, as entrevistas foram efetivadas.

### Análise dos resultados e estatística

A análise dos dados foi realizada em duas etapas: 1) a validade baseada na estrutura interna, a qual está relacionada às análises estatísticas considerando modelos para medidas de traço latente. Nesta etapa, utilizou-se a análise fatorial exploratória (AFE) e confirmatória (AFC); e 2) validade baseada no padrão de resposta aos itens, a qual se refere às avaliações teóricas e empíricas sobre a forma como os participantes responderam ao instrumento. Para este estudo, utilizou-se análises baseadas na teoria de resposta ao item como o *differential item functioning* ^(11)^.

Para realização da AFE, inicialmente, foram calculadas as medidas de adequação amostral do instrumento através do KMO e, posteriormente, foram calculadas a MSA por item^(11)^. Para o teste de esfericidade de Bartlett, adotou-se valores de p-valor ≤ 0,05^(12)^. O método utilizado para realizar a AFE foi o da análise fatorial policórica, denominada *Weighted Least Squares* (WLS – Mínimos Quadrados Ponderados). A consistência interna foi verificada através do Alfa de Cronbach, com valores iguais ou acima de 0,70 para o instrumento ser considerado preciso/fidedigno^(13)^.

Os protocolos seguidos para a interpretação dos resultados da AFE foram os seguintes índices e valores: a comunalidade (h^2^), referente à medida da relação inter-itens; o valor próprio, utilizado como critério para definir o número de fatores^(14)^; o gráfico de escarpa, conhecido como *screeplot,* interpretado a partir do critério de Cattell, também utilizado para definir o número de fatores^(15)^; e a carga fatorial dos itens, medida com o objetivo de verificar quais itens estão alocados em quais fatores, adotou-se para a carga fatorial o valor de ±0,30^(11)^.

Para a realização da AFC, utilizou-se os princípios da modelagem de equações estruturais (MEE). Foram analisados os seguintes indicadores: χ^2^ (qui-quadrado); *Comparative Fit Index* (CFI) e *Tucker-Lewis Index* (TLI); Godness-of-Fit Index (GFI) e o Adjusted Goodness-of-Fit Index (AGFI); Root-Mean-Square Error of Approximation (RMSEA)^(16,17)^.

Para o χ^2^ considerou-se 3 como valor máximo para indicar que o modelo teórico está ajustado aos dados. Para o CFI e TLI foram considerados escores superiores a 0,90 para afirmar que o modelo pretendido teoricamente representa da melhor forma o construto. Para o GFI e AGFI, utilizou-se valores superiores a 0,90 e para o RMSEA, valores ≥ 0,05, observa-se, no entanto, que para amostras maiores aceita-se o valor 0,08^(16,17)^.

Para analisar a validade baseada no padrão de resposta aos itens, aplicou-se a teoria de resposta ao item (TRI). No caso deste estudo, os dados são politômicos ou categóricos, sendo assim é necessário aplicar um modelo para respostas graduais, de modo que, no caso deste estudo, aplicou-se o modelo de resposta gradual de Samejima que investiga a discriminação, que variam de 0 a +3, sendo quanto maior, mais discriminação e a dificuldade em escalas ordinais ou categóricas, que vão de -3 a +3, sendo mais fáceis e mais difíceis, respectivamente^(18,19)^.

## RESULTADOS

Os resultados serão apresentados respeitando as seguintes etapas: análise fatorial exploratória, análise fatorial confirmatória e análise de ajuste dos itens – teoria de resposta ao item.

### Análise Fatorial Exploratória

A avaliação da adequação amostral mostrou resultados satisfatórios (KMO=0,786). O Teste de Esfericidade de Bartlett também apresentou resultado significativo [χ^2^(62) = 2446.8, p-valor<0,001]. A adequação dos itens verificada pelo KMO apresentou resultados superiores a 0,50, exceto para os itens: “Já chegou a faltar preservativo masculino na unidade?”(MSA = 0,498);“Já chegou a faltar preservativo feminino na unidade?”(MSA = 0,491);“Ocorre na Unidade de Saúde da Família o atendimento para a realização da profilaxia pré-exposição (PrEP)?” (MSA = 0,483); e “Ocorre na Unidade de Saúde da Família o atendimento para a realização da profilaxia pós-exposição (PEP)?” (MSA = 0,483). Contudo, por considerar esses valores marginais, optou-se pela sua manutenção nas análises seguintes.

Na realização da análise fatorial pelo método WLS, verifica-se que através do critério Kaiser, baseado no número de *Eigenvalues* (valores próprios), sugeriu-se 19 fatores, no critério Cattell, baseado no ponto de cotovelo, identificado no gráfico como *Optimal Coordinates*, indicou-se 9 fatores. O critério da *Parallel Analysis* (análise paralela) é o critério de Horn, que também indicou 9 fatores, já o critério *Acceleration Factor* apontou para o número de 2 fatores para o instrumento (Figura 1).

**Figura 1:** Gráfico comparativo para escolha do número de fatores para o instrumento, Recife, Pernambuco, Brasil, 2022

No processo de decisão acerca dos fatores, considerando-se suas cargas fatoriais, foram realizadas AFEs para estruturas contendo 9 fatores, conforme indicado pelos métodos da análise paralela e coordenada ideal – *Optimal Coordinates* – assim como foi para a estrutura bifatorial conforme indicado pelo fator de aceleração. Contudo, as duas estruturas foram insatisfatórias em termos teóricos. Dessa forma, testou-se mais duas versões com 7 fatores sugeridos pelos juízes especialistas e com 5 fatores, sugeridos pelos revisores técnicos, na validação de conteúdo^(7)^.

Acerca das análises realizadas entre fatores e itens, afirma-se que o item pertence a determinado fator se apresentar valores entre -0,3 e ≥0,3^(20,21)^. Quando distribuídos os fatores e seus respectivos itens, observou-se que na opção com 9 fatores, apenas 4 itens não fatoraram. Na opção de 7 fatores, 3 itens não fatoraram. Já na opção com 5 fatores, 8 itens não fatoraram, e com 2 fatores, 16 itens não fatoraram.

Julgou-se que a estrutura que apresentou melhor adequação considerando-se termos estatísticos e teóricos foi a estrutura que apresenta 5 fatores. Assim, os fatores foram renomeados como WLS1 – Vigilância, prevenção e Redes de Atenção à Saúde; WLS2 – Apoio, diagnóstico e acolhimento; WLS3 – Educação em saúde; WLS4 – Organização da assistência; e WLS5 – Recursos físicos, materiais e humanos e os itens relocados de acordo com cada fator.

Apesar dos itens 1, 4, 5, 6, 7, 19, 52 e 54 não apresentarem resultados iniciais dentro das recomendações estatísticas, optou-se pela permanência dos itens no instrumento por questões teóricas. Posteriormente, os itens foram relocados para o fator que melhor se enquadrassem, exceto os itens 19 e 54 que foram retirados da análise, após considerações acerca das suas cargas fatoriais e do seu suporte ao modelo estatístico. Ressalta-se que apesar de os itens 19 e 54 não fatorarem, estes não foram excluídos, devido à importância teórica, sugerindo-se a aplicação destes itens em estudos futuros. Sendo assim, o item 19 foi alocado para o fator WLS5 – Recursos físicos, materiais e humanos e o item 54 para o fator WLS4 – Organização da assistência.

### Análise Fatorial Confirmatória

De acordo com os resultados, o instrumento possui valores indicativos da confiabilidade composta (0,99) e da variância média extraída (0,99) em todas as associações realizadas quanto aos fatores e seus respectivos itens. Além disso, evidencia-se que, para todas as associações, o p-valor foi significativo (p-valor<0,05), sendo suficiente para considerar este instrumento válido e preciso. Os indicadores de ajuste da Modelagem de Equações Estruturais (MEE) para a validação do instrumento estão apresentados na Tabela 1. Os critérios seguem a interpretação explicada na metodologia, referente aos indicadores necessários para o ajuste do modelo aos dados. Considera-se que a estrutura do instrumento é válida de acordo com os critérios indicativos. Reforça-se que os itens 19 e 54 não foram considerados para a realização da AFC, tendo em vista as decisões tomadas quanto ao suporte estatístico e teórico para a manutenção destes itens.

**Tabela 1:** Indicadores de ajuste da Análise Fatorial Confirmatória para validação de constructo do instrumento de avaliação do processo de descentralização do atendimento a Pessoas Vivendo com HIV para Atenção Primária à Saúde, Recife, Pernambuco, Brasil, 2022

Indicador de ajuste	Critérios para bom ajuste do modelo	Modelo final
Ajuste absoluto		
Função de discrepância: χ^2^ (valor p)	-	2749,959 (0,001)
Qui-quadrado normado (χ^2^/gl)	Valor entre 1 e 5	1,56
GFI (índice de qualidade de ajuste)	Acima de 0,90	0,90
AGFI (índice de qualidade de ajuste ajustado)	Acima de 0,90	0,90
RMSEA (raiz média quadrática dos erros de aproximação)	Entre (0,05;0,10] p(H^0^: RMSEA≤0,05)	0,047
Ajuste relativo		
TLI (índice Tukey-Lewis)	≥0,90	0,915
CFI (índice de ajuste comparativo)	≥0,90	0,918

### Análise de Ajuste dos Itens – Teoria de Resposta ao Item

O modelo das análises foi decidido por meio do teste ANOVA realizando a comparação entre os modelos *Generalized Partial Scale Model* ^(22)^ e Modelo de Respostas Graduais de Samejima^(19)^. Segundo os resultados, o modelo escolhido foi o modelo de respostas graduais de Samejima com resultado da ANOVA significativo [χ^2^(0) = 216.078; p-valor < 0,01].

Destaca-se que, neste caso, não se espera valores significativos para o p-valor. Apesar dos itens 19 e 54 não terem sido considerados na AFC, eles apresentaram bons índices de ajuste, podendo ser mantidos no instrumento para pesquisas posteriores. Em contrapartida, os itens “A Unidade de Saúde da Família oferta teste rápido para HIV para todas as gestantes em acompanhamento na unidade?” (p-valor =0,030); “A Unidade de Saúde da Família disponibiliza teste rápido para HIV para indivíduos diagnosticados com tuberculose?” (p-valor=0,000); “A Unidade de Saúde da Família disponibiliza de medicamentos antirretrovirais para o tratamento do HIV?” (p-valor=0,043); e o item “As Unidades Dispensadoras de Medicamentos (UDM) atendem os pacientes com HIV referenciados pela Unidade de Saúde da Família?” (p-valor=0,28), apresentaram resultados significativos quanto ao p-valor, o que indica que esses itens não se encontram ajustados para a validação com base nos parâmetros dos itens. Observa-se, ainda, que o valor de RMSEA foi inferior a 0,05 para todos os itens, exceto o item 40 (0,07) mesmo assim inferior a 0,08.

Sendo assim, a indicação para a maioria absoluta dos itens é de ajuste adequado. Desse modo, em sua versão final, os 63 itens foram organizados, de acordo com seu fator. Cada item deve ser avaliado utilizando uma escala do tipo *Likert*, com as seguintes opções: Nunca, Algumas vezes, Frequentemente, Muito frequentemente e Sempre (Figura 2).

**Figura 2:** Versão final do instrumento de avaliação do processo de descentralização da assistência à Pessoas Vivendo com HIV na Atenção Primária à Saúde, com cinco fatores e sessenta e três itens, Recife, Pernambuco, Brasil, 2022

## DISCUSSÃO

A validação de constructo, considerada a principal medida de avaliação de um instrumento, foi realizada pela AFE, AFC e análise do ajuste aos itens, com a TRI^(23,24)^. Destaca-se a relevância da utilização do conhecimento científico para construção de instrumentos confiáveis para tomada de decisão em serviços de saúde. A adoção desses instrumentos impulsiona os ganhos em qualidade e possibilitam a melhoria da assistência às pessoas vivendo com HIV na Atenção Primária à Saúde.

Com isso, a mensuração das ações tomadas pelo gestor em prol da melhoria do processo se torna mais efetiva. Assim, a utilização do instrumento pelas equipes multidisciplinares e pelos gestores proporciona a cooperação entre si para o atingimento de um determinado objetivo. Além disso, ela possibilita o pensamento sistêmico sobre a rede de cuidados às pessoas vivendo com HIV e auxilia os profissionais, os gestores e os indivíduos a perceberem que todos fazem parte de um único sistema que busca a melhoria continua no modelo de cuidado integral às PVHIV^(6,7,8)^.

Os resultados da análise do instrumento mostraram-se satisfatórios acerca da adequação da amostra e da matriz e correlações/ covariância para quase todos os itens, exceto para quatro itens, que não foram excluídos, uma vez que todos os profissionais de saúde participantes declararam “nunca” como resposta para os itens, isso é, nunca chegou a faltar preservativo masculino e feminino nas unidades investigadas. Além disso, os itens se encontram dentro das recomendações do Ministério da Saúde e, por esse motivo, optou-se pela não exclusão dos itens. Os itens que tratam da disponibilização da Profilaxia Pré-Exposição (PrEP) e Profilaxia Pós-exposição (PEP) também foram mantidos por configurar ações que devem ser realizadas na APS^(25)^.

Na proposta do novo modelo de cuidado integral às pessoas vivendo com HIV, destaca-se o conceito de prevenção combinada, que se configura como um conjunto de medidas comportamentais, biomédicas, estruturais e envolve diferentes níveis de atenção. Assim, o uso do preservativo masculino e feminino revela-se como o principal método de barreira para a prevenção de HIV e das ISTs, sendo um método barato, de fácil acesso e eficaz, recomendando-se a oferta do insumo de forma ampla e sem barreiras de acesso^(26,27)^.

Atualmente a PrEP é considerada uma das estratégias mais inovadoras de prevenção ao HIV, devendo ser amplamente difundida na APS. Verifica-se que a aceitação da PrEP ainda é baixa, devido a algumas barreiras encontradas nesse nível de atenção, como: falta de financiamento, dificuldade de acesso, estigma, ausência de treinamentos e de conhecimento por parte dos profissionais de saúde, dentre outros^(28,29)^. A descentralização do atendimento e a prescrição da PrEP pode ser uma alternativa para a ampliação. Em um estudo recente, realizado no Canadá, verificou-se o aumento da adesão à medida quando realizados por enfermeiros^(30)^. A PEP é outra estratégia recomendada, seu uso vem aumentando cada vez mais no Brasil e no mundo, sendo recomendada em situações de acidentes com materiais biológicos, violência sexual, assim como na exposição sexual consentida^(31)^.

Verifica-se que a opção de 5 fatores não foi a alternativa que apresentou o menor número de itens que não fatoraram, mas a escolha dessa opção se deu pelo fato de que os itens se elencaram com aspectos semelhantes. A AFE tem como objetivo determinar o número variáveis latentes (fatores) que melhor representam um número de variáveis observadas (itens), isto é, as variáveis observadas são determinadas a um mesmo fator quando possuem variância em comum^(32)^. A AFE exige dos pesquisadores uma série de decisões para que se possa obter uma estrutura fatorial adequada (a relação do fator com os itens), sendo necessário que tais decisões estejam ancoradas em critérios teóricos e metodológicos^(14)^.

Acerca dos itens que não fatoraram, evidencia-se que os itens 1 e 4 estão relacionados à disposição de salas para o atendimento individualizado realizado por profissionais de saúde de nível superior; os itens 5, 6, 7, 8 e 19 exploram aspectos voltados à presença, ausência e acesso a insumos, tais como os testes rápidos e preservativos. Os itens 52 e 54 investigam as capacitações de profissionais acerca da realização da testagem rápida e orientação quanto ao uso dos ARV.

Desse modo, recomenda-se a disponibilidade de espaços físicos adequados que garantam o sigilo e a privacidade dos pacientes nos atendimentos voltados ao acolhimento, aconselhamento, testagem, atividades de educação em saúde, dentre outros^(27)^. Ressalta-se que as práticas voltadas a educação em saúde vão além da transmissão de conhecimento, essas práticas devem promover o empoderamento do indivíduo e da população, uma vez que através da informação é possível a mudança de atitude e a adoção de práticas mais consistentes com as estratégias de prevenção às IST/HIV/Aids^(33,34)^. Pontua-se que os profissionais de saúde que irão compor a RAS às PVHIV devem dispor de apoio matricial pelos SAE, além disso, recomenda-se que tais profissionais sejam capacitados através de estágios supervisionados, consultas compartilhadas, rodas de conversa, por exemplo^(1,29)^.

A respeito da AFC, verifica-se que o instrumento apresentou níveis de confiabilidade composta e variância média satisfatórias. Sobre os indicadores de ajustes da AFC, observa-se que o instrumento respeitou os critérios para bom ajuste do modelo, com RMSEA, GFI, CFI satisfatórios. O índice de ajuste são medidas que auxiliam na verificação de quão bom o instrumento se encontra e, neste caso, o instrumento se enquadrou em todos os critérios, sendo considerado validado^(9)^.

Destaca-se que a descentralização do atendimento às PVHIV para APS é uma possibilidade que proporcionaria a ampliação do acesso às PVHIV aos serviços de saúde, com o aumento da testagem, do diagnóstico, do início do tratamento em tempo oportuno, assim como a prevenção da infecção pelo HIV, através da utilização da PrEP e PEP. Entretanto, evidencia-se que ainda há vários entraves para o êxito da proposta^(35,36)^, como a falta de estrutura física para execução do aconselhamento e testagem rápida, ausência de capacitação profissional, materiais e insumos, como por exemplo, materiais educativos, tais como: folders, cartazes, camisinha feminina, o que reflete negativamente na qualidade da assistência^(37)^.

Diante deste contexto, observa-se que, apesar das recomendações do Ministério da Saúde para a descentralização do atendimento às pessoas vivendo com HIV para a APS, faz-se necessário maiores investimentos nesse nível de atenção, com estruturação das unidades, definição de fluxos de acesso, oferta de materiais e insumos, garantia de recursos humanos capacitados e em número adequado. Observa-se na APS algumas ações estratégicas adotadas, tais como: atividades voltadas para o acolhimento e aconselhamento, oferta de testagem rápida e de exames sorológico para HIV, sífilis e hepatites virais, disponibilização de preservativos e géis lubrificantes, busca ativa, notificação e práticas de educação em saúde^(35,36,37,38,39)^ que pode ser o caminho para a implantação integral do novo modelo de atenção às pessoas vivendo com HIV.

Instrumentos de avaliação da qualidade da gestão em saúde são a base cíclica da dinâmica da gestão para melhoria dos processos. Permitem os ciclos de aprendizagem e a implantação de planos de ação ou de padronização de práticas de forma sistêmica e com a participação da equipe multiprofissional. Para maior efetividade na aplicação do instrumento, é preciso considerar a transformação digital em saúde e a adesão a tecnologias que possibilitem maior eficiência no atendimento, redução de custos, padronização de qualidade da estrutura físico funcional, dos processos e dos resultados. A incorporação do presente instrumento de avaliação em plataformas que podem ser desenvolvidas em pesquisas futuras, ou já existentes na APS, agilizam os processos de avaliação da gestão em saúde e contribui para a tomada de decisões de maneira mais rápida e eficaz.

### Limitações do estudo

O estudo apresenta como limitação o fato de ter sido desenvolvido em apenas um município do Nordeste. Além disso, a amostra coletada foi para realização da validação constructo, sendo, ainda, recomendada a aplicação do estudo em amostra maiores, inclusive em outros serviços para possibilitar a validação dos escores referentes aos fatores.

### Contribuições para as áreas da enfermagem, saúde ou políticas públicas

Espera-se que o instrumento validado contribua na avaliação do processo de descentralização da assistência às PVHIV na APS e na estruturação/reestruturação de unidades, uma vez que a APS possui potencial para a realização deste atendimento. Além de contribuir com os profissionais de saúde, com destaque para enfermeiros que atuam na assistência à PVHIV, na verificação de aspectos essenciais para a implantação/consolidação da proposta do novo modelo de cuidado às pessoas vivendo com HIV.

## CONCLUSÕES

O instrumento de avaliação da assistência a Pessoas Vivendo com HIV para Atenção Primária à Saúde encontra-se validado quanto ao constructo de acordo com as recomendações preconizadas pelas instituições AERA, APA e NCME. Possui em sua versão final 63 itens, distribuídos em 5 fatores, sendo eles: Organização da assistência; Apoio, Diagnóstico e Acolhimento; Educação em Saúde; Vigilância, Prevenção e Redes de Atenção à Saúde; Recursos Físicos, Materiais e Humanos, sendo avaliado a partir de uma escala do tipo *Likert*.

Espera-se que o instrumento auxilie na avaliação em saúde, bem como na estruturação e/ou reestruturação de unidades de saúde com vistas ao atendimento integral e de qualidade às PVHIV na APS. Além disso, acredita-se que o uso deste instrumento possibilitará a avaliação da implantação das ações voltadas ao processo de descentralização do atendimento às PVHIV para APS, como também à identificação das potencialidades e fragilidades do processo, com vistas à qualidade da assistência e à integralidade do cuidado às PVHIV. Recomenda-se a condução de novos estudos com objetivo de realizar a normatização dos escores do instrumento validado, bem como a incorporação do instrumento em plataformas digitais.
